# Obesity in Italy: An Empirical Analysis of Healthcare Consumption, Quality of Life and Comorbidities

**DOI:** 10.3390/medicina61061061

**Published:** 2025-06-09

**Authors:** Elenka Brenna, Claudio Jommi

**Affiliations:** Department of Pharmaceutical Sciences, Università degli Studi del Piemonte Orientale “Amedeo Avogadro”, Largo Donegani 2, 28100 Novara, Italy; claudio.jommi@uniupo.it

**Keywords:** obesity, Italy, socioeconomic drivers, propensity score matching, quality of life, comorbidities, healthcare consumption

## Abstract

*Background and Objectives*: Obesity is a health condition that significantly augments the risk of several chronic diseases and is a major public health concern. In Italy, this phenomenon has highly increased in the last few decades, raising alarm about both NHS sustainability and population health. We investigate whether and to what extent obesity impacts three different categories of outcomes, namely healthcare consumption, quality of life and the presence of relevant comorbidities. *Materials and Methods*: We use data from the European Health Interview Survey, 2019, a micro dataset that displays more than 45,000 observations, providing detailed information on the health status and healthcare access, demographics and socioeconomic characteristics among Italian individuals aged 15 and over. We first provide a descriptive analysis of the socioeconomic gradient of obesity, and then we implement propensity score matching to detect the effects of this condition on healthcare use, quality of life and comorbidities. To the best of our knowledge, this is the first empirical paper to jointly consider the healthcare consumption, declared comorbidities and quality of life of Italian obese individuals. *Results*: The findings show that obese individuals consume more health services, have a lower quality of life and present a higher rate of comorbidities compared to non-obese individuals with the same observable characteristics. We also find that obesity is rooted in socioeconomic drivers, with richer and more educated individuals being less likely to present with this condition. *Conclusions*: The findings call for policy measures aimed at monitoring and contrasting the rising phenomenon of obesity in Italy, with a tailored approach across socioeconomic groups.

## 1. Introduction

Obesity is a health condition that significantly increases the risk of several chronic diseases, including cardiovascular diseases, type 2 diabetes mellitus, cancers, chronic kidney disease, musculoskeletal disorders, gastrointestinal tract diseases, respiratory diseases, and depression [1,2]. Its prevalence is steadily intensifying worldwide [3] and Italy follows this trend, with a rise of almost 30% in the adult obese population in the last three decades [4].

Numerous studies have investigated the economic burden of obesity and its implications from a societal viewpoint. Obesity increases healthcare costs, implies indirect costs in terms of absenteeism and productivity loss [5] and reduces both individuals’ quality of life [6,7] and their self-esteem [8,9].

The effects of obesity on healthcare consumption are well represented by the literature on industrialized countries. According to recent reviews, the costs vary along with different factors, namely the definition of obesity, the specific country, the eventual inclusion of overweight together with obesity, the main perspective (whether an individual or societal standpoint) and the assessment of premature mortality [10,11]. When also including related diseases (cardiovascular diseases, diabetes mellitus and cancers) and other possible direct costs attributable to this condition (e.g., those related to bariatric surgery), healthcare expenditure highly increases, so much so that some authors suggest that this condition outranks both smoking and drinking in its harmful impact on health and healthcare costs [12].

Together with the societal costs of this condition, there is a growing body of literature considering the quality of life of obese individuals and the possible public measures aimed at correcting lifestyles, such as modifying eating and physical activity habits [13]. Both physical and psychosocial functioning are negatively affected by excess weight: impairment in daily activities has long been recognized as well as the negative effects on self-esteem, anxiety and depression [14,15]. Several studies investigated the condition of obesity as a risk factor for different kinds of disease; in particular, diabetes, hypertension, coronary heart disease, stroke, respiratory problems, sleep apnea, osteoarthritis, and some cancers [16]. These comorbidities are a cause of concern due to the high impact on both national healthcare costs and individuals’ health status and quality of life.

The multifaceted aspects related to obesity suggest that this condition requires constant monitoring and a broad evaluation across multiple domains, including health outcomes, impairment in daily activities, and economic evaluation.

With reference to Italy, the extant literature is mainly focused on the economic burden of obesity. A recent contribution by [4] reports that the whole costs related to this condition amounted to EUR 13.34 billion in 2020 (0.8% of Italian GDP), with the direct costs representing 59% of the total resources (4.8% of national healthcare expenditure). These costs are mainly attributable to cardiovascular diseases, diabetes, cancers, and bariatric surgery. Presenteeism (presence at the workplace in conditions compromised by the disorder) and work absenteeism accounted for 52% and 48% of the total indirect costs, respectively. A different approach is provided by [17], who exploit the longitudinal nature of the data to analyze the lifetime health-related costs attributable to the condition of obesity. Their findings show that obesity represents a cause of concern due to the high differential in resource absorption across the whole life of obese individuals. Other contributions from Italy concentrate their analysis exclusively on the costs related to selected healthcare services, relying on administrative databases [18,19].

The abovementioned studies are focused on the economic impact of obesity and do not provide relevant information on the obese population in terms of the socioeconomic characteristics, declared comorbidities, and quality of life.

We want to fill this gap through the adoption of a multifactorial perspective and the use of updated microdata representative of the Italian population, as drawn from the European Health Interview Survey (EHIS), 2019 [20]. We first study the distribution of obesity according to socioeconomic characteristics to highlight the unequal probability of developing this condition across the Italian population. We then implement propensity score matching (PSM) to test the impact of obesity on three different classes of outcomes, namely healthcare consumption, quality of life and the presence of comorbidities. Although worldwide several studies have analyzed the impact of this condition on selected outcomes, this is the first attempt to inspect different aspects related to obesity using data representative of the Italian population. Obesity is a relatively recent phenomenon in Italy, which raises concern about both NHS costs and population health. Our results offer insights for policy measures able to tackle the increasing trend of this condition across the Italian population.

## 2. Materials and Methods

The empirical analysis is performed on the Italian database drawn from the European Health Interview Survey [20]. The questionnaire was administrated by the Italian National Office of Statistics between September and December 2019. The dataset displays more than 45,000 observations, providing detailed information on the health status and healthcare access, demographics and socioeconomic characteristics among Italian individuals aged 15 and over. It involves approximately 30,000 households, across 835 Italian municipalities of very heterogeneous population size. After cleaning missing values, the final sample consists of 44,902 observations.

### 2.1. Obesity in Italy: The Socioeconomic Gradient

**Figure 1:**
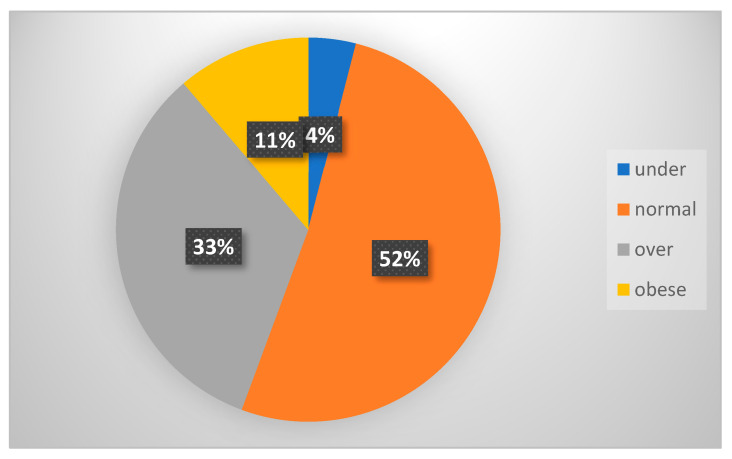
Distribution of BMI classes among the Italian population—year 2019. Source: Our elaboration based on the EHIS, 2019 [20].

Data on body mass index are self-reported; in the survey, respondents are asked to report their height without shoes and their weight without clothes. With this information, the interviewer calculates the body mass index and clusters it according to four possible categories: underweight, normal weight, overweight and obese (the obese cluster is not further split into the three levels of obesity). The percentage of obese people is 11.1%, while individuals who are overweight represent 33% of the population, as shown in Figure 1.

**Figure 2:**
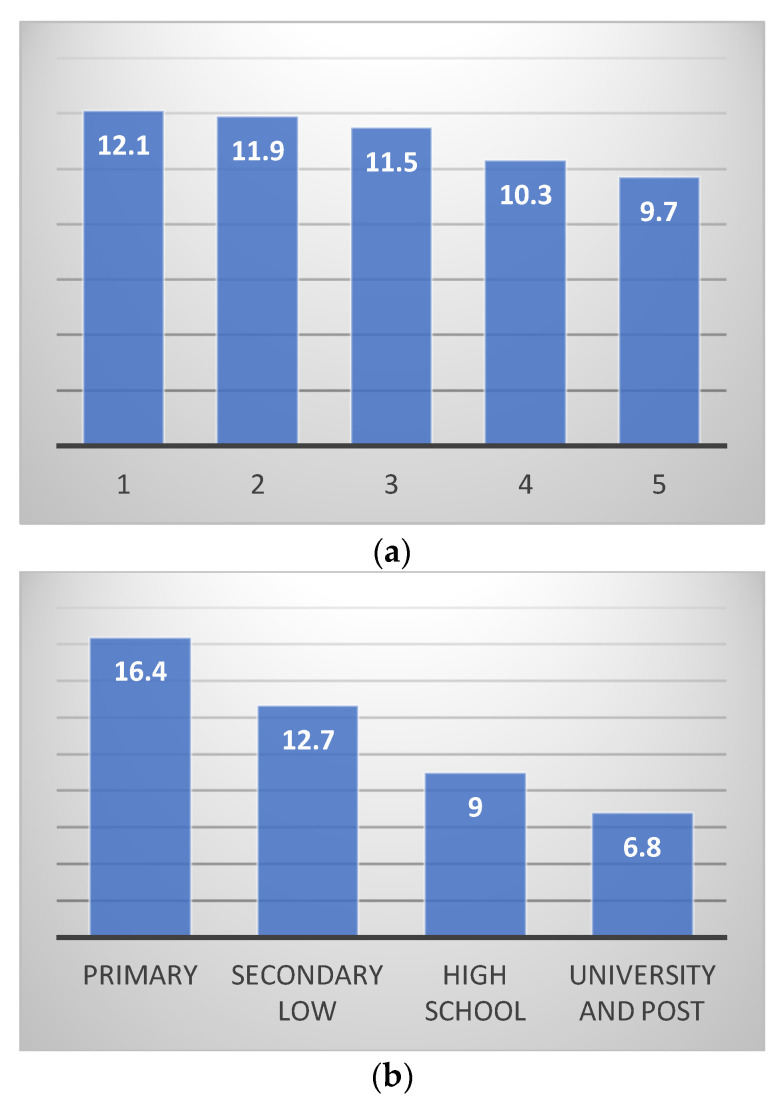
(**a**) Distribution of obesity according to income quintiles—percentage values. Income quintiles from poorest (1) to richest (5). (**b**) Distribution of obesity according to educational level—percentage values. Source: Our elaboration based on the EHIS, 2019 [20].

Since obesity is highly related to lifestyle and healthy habits are driven by socioeconomic factors [21], we investigated the distribution of obesity according to both income and education. Evidence from developed countries shows that individuals belonging to higher social classes and who are better qualified are less likely to be obese [22,23,24]. Figure 2a,b displays the distribution of individuals who are obese according to both income quintiles and educational levels. The two histograms confirm a socioeconomic gradient for obesity: specifically, a gap of almost 2.5 percentage points is detected among the richest and the poorest share of the population; as for education, the share of obese individuals is 16.4% among people with primary education versus a 6.8% value for individuals holding a university or post-university degree. This evidence, however, is to be read with caution due to a possible “generational effect” in this distribution. Older individuals are more likely to have just primary education compared to the youngest cohorts and elderly people are more numerous and at a higher risk of obesity than younger generations. This possible bias will be solved when implementing stronger empirical methods, as shown in the section dedicated to the model specification.

We also find a north–south divide in the distribution of obesity, with a higher percentage of obese people in the south of Italy (13%) compared to the north (10%) and center of Italy (10%). This evidence could be explained by different lifestyles, possibly related to heterogeneous socioeconomic conditions at different latitudes in Italy. On average, in the southern regions, the level of education is lower compared to the northern regions and there is a higher presence of people on a low income.

### 2.2. Model Specification: The Propensity Score Matching

The aim of this study consists of investigating whether being obese may (i) increase the use of healthcare services; (ii) affect the quality of life; and (iii) represent a risk factor for the presence of other diseases. In analyzing the effects of obesity on the selected outcomes, a problem of endogeneity due to selection bias may arise; for example, if we want to test whether obesity impairs quality of life, causality is hard to disentangle, as individuals with difficulties in daily life activities are more inclined to a sedentary life and this may increase the risk of obesity. As suggested by [25], one possible solution to the selection problem is represented by PSM. This method is extensively used in empirical analysis in the field of social and health science [26,27,28] and specifically adopted to address endogeneity in studies investigating obesity [29]. Through this technique, it is possible to identify two different groups of individuals showing exactly the same observable characteristics except for the condition of obesity. Eventual differences in the probability of showing a given outcome are then to be attributed to the presence of obesity [30].

The identification process is performed by matching each obese individual with their non-obese “statistical twins”, showing the same traits but for the condition of obesity. By pairing the two categories of individuals, it is possible to control for a series of confounding factors that may affect the final outcome [31,32]. According to both the literature suggestions [33,34] and data availability, we create a vector X of confounding factors, including demographic, socioeconomic, lifestyle, health and healthcare access characteristics. Once all these variables are controlled for, the eventual differences between the two groups in the final outcomes (i.e., variables addressing either healthcare use, the presence of chronic conditions or quality of life) are to be related to the unique difference, which is represented by the condition of obesity.

In running the PSM, an assumption is made, namely that the assignment into the treatment (in this case, the obesity condition) is exclusively attributable to the observable covariates. This is called the “conditional independence assumption”, as addressed by [35]; we have at disposal a rich set of variables, and this allows us to rely on the given hypothesis in our specification.

Technically, PSM is performed through different steps. First, a probit is run, which investigates the probability of being obese given the vector X of the covariates. The outcome variable is a binary variable taking a value of one if the individual is obese and zero otherwise. The parameters resulting from the probit are transformed into a score for the matching procedure, which is able to select two groups of individuals identical in all the observable traits and differing solely for the condition of obesity. Our sample presents a rather small number of treated (*n* = 4951) compared to untreated individuals (*n* = 39,951). In the presence of relevant differences in size between the two groups, radius matching is considered an efficient technique due to its faculty for matching each treatment unit i with multiple comparison units within a defined band, thus avoiding the risk of bad matching [25,36]. We employ radius matching with caliper 0.01. In running the PSM, we impose the common support, which ensures that individuals with the same X characteristics have the same probability of participating in both the treated and untreated group [37].

As a third step, the average treatment effect on the treated (ATT) is measured: it represents the average difference, among the treated and untreated, in the probability of showing the outcome variables. In a formula, it is expressed as:

$$E\left(Y_{1}-Y_{0}\right)|\;W=1$$

where

W = treatment (condition of obesity)
Y_1_ = binary outcome variable for the treated
Y_0_ = binary outcome variable for the untreated

The outcome variables for both the treated and untreated are binary variables assuming a value of one if a certain condition is encountered, and zero otherwise (see the next section). The quality of PSM is finally checked through the covariate balancing *t*-test, which measures the bias reduction after the matching for each of the x values.

The PSM is run using Stata 13 and through the program psmatch2, set up by [38].

#### 2.2.1. Variable Selection and Descriptive Statistics: Dependent Variables

Our specification entails the presence of several dependent variables addressing three different domains, namely healthcare use, quality of life and the presence of other diseases. Each outcome variable is a binary variable assuming the value of one if a condition is revealed and zero otherwise. To build a binary variable, we exploit the information provided by the survey. For example, from the question “*During the last two weeks, did you consume pharmaceutical products prescribed by a doctor?*”, we create a binary variable assuming value of one if the answer is positive and zero otherwise.

The description of each dependent variable and its mean distribution across the three samples, respectively, the full sample (*n* = 44,902), the sample of treated (*n* = 4951) and the sample of untreated (*n* = 39,951), is reported in Table 1. Descriptive statistics reveal the unequal distribution of the variables between the treated and untreated groups. On average, obese individuals show a higher consumption of healthcare services compared to non-obese individuals, with the exception of the use of drugs not prescribed by a doctor. High differentials are shown in the access to physicians; for example, obese individuals show a value of 17.4% for two or more visits to a general practitioner (GP) in the four weeks preceding the interview versus a value of 10% among untreated individuals. Similarly, evidence on the presence of comorbidities shows that obese individuals are more likely to report the selected diseases: 18% of obese individuals have diabetes versus 6% among non-obese individuals, while the rate of hypertension among the treated group (42%) is more than double its counterfactual (20%), and this proportion is held for almost all the other comorbidities. High differences are also revealed for the variables addressing the quality of life; for example, among obese individuals, 23% show problems in walking up or down 10 steps versus 10% among non-obese individuals. The descriptive analysis of the outcome variables supports our research questions: we found higher values for healthcare consumption, higher prevalence of comorbidities and more difficulties in daily life actions for the treated compared to the untreated group.

#### 2.2.2. Variable Selection and Descriptive Statistics: Independent Variables

With reference to the choice of independent variables, the selection was subject to the conditional independence assumption: we had to choose variables related to the treatment (condition of obesity) and considered good predictors of the outcomes. According to the data availability and the literature suggestions, we selected a set of confounding factors from demographics, socioeconomics, health status and healthcare access. We were conscious that the selected variables could not include all the variables with a possible impact on obesity. However, we had at our disposal a rich set of relevant variables, which made us rely on the conditional independence assumption [35]. Table 2 reports the whole set of chosen dependent variables. In terms of this selection process, a clarification is necessary: in running the PSM on each of the three selected outcomes, we had to exclude eventual covariates that were represented in the set of dependent variables. For example, in the model testing whether obese individuals consume more healthcare services compared to their “statistical twins”, we excluded from the covariates visits to both a GP and a specialist, because they represented the outcome. More insights on the model specification are provided in Section 3.

The results derived from the descriptive statistics, together with the definitions of the variables, are reported in Table 2. With the exception of the income quintiles, all the selected variables are binary variables, describing a given characteristic. The variables addressing health status are represented by the presence of chronic conditions and severe limitations and by self-assessed health. With reference to the latter, respondents were asked how would they rate their own health, with five possible answers: *very well*, *well*, *fine*, *bad*, *very bad*. We collapsed the categories at the edges and created three binary variables, respectively, *good*, *fine* and *bad health*. Healthcare use is represented by visits to the GP, specialists and psychiatric/psychologist, and by access to diagnostic services. The waiting times provide some more evidence on access to care: respondents were asked whether they experienced delays in access to healthcare due to long waiting times, with possible answers of yes or no; we created a dependent variable assuming a value of one for the positive answers and zero otherwise. Socioeconomic characteristics are represented by working status, educational level and income quintiles. Education is strictly correlated with the probability of experiencing obesity: we created three binary variables, the first one identifying all the individuals who finished lower secondary school, the second one addressing individuals with a high school diploma (omitted variable) and the third one indicating people holding a university or post-university degree. As age is a strong predictor of obesity, we decided to keep all the eleven age classes proposed by the EHIS and to omit the age class ranging from 35 to 44 years. Geographical variables were included to control for fixed effects related to different lifestyles at different latitudes. Healthy/unhealthy habits provide more insight into individual lifestyle.

The covariates show a different distribution across the two classes (obese and non-obese), corroborating the literature findings; obesity depends on socioeconomic factors, especially education (10% of obese individuals hold a university or post-university degree vs. 16% among non-obese individuals); employed individuals are less represented among the obese (38% vs. 43%); age is a predictor of this condition and obese individuals are more likely to experience severe limitations (14% vs. 7%) and chronic conditions (50% vs. 30%) and to rate their health status as bad/very bad (15% vs. 8%).

## 3. Results

In what follows, we first present the results of the pre-matching phase on which the propensity score is built; subsequently, we show the results from the PSM; and finally, we provide evidence on the quality of the matching via a balancing *t*-test.

### 3.1. Pre-Matching Probit and Description of Independent Variables

The first step in PSM consists of running a probit, which allows us to identify the effects of all the covariates on the probability of being obese.

The results from the pre-matching probit corroborate the findings from the descriptive analysis and confirm that this condition is rooted in socioeconomic factors (due to the editorial rules, the number of tables is limited; the results are available from the corresponding author). Specifically, income and education are inversely related to the probability of being obese. The working status (employed, unemployed, retired or other) does not show any significance. Reporting bad or fine health, as opposed to good health, as well as the presence of limitations and chronic conditions, is a good predictor of the outcome. For healthcare use, the data shows that obese people are more likely to have visited the GP in the last four weeks, while no significance is shown for specialist visits. This evidence suggests that GPs have a role in managing this condition and orienting the patient in his/her treatments [39]. Having experienced long waiting times in terms of the access to healthcare is related to the probability of being obese. For demographics, age is a good predictor of the outcome, with individuals belonging to age classes inferior to the omitted variable (age between 35 and 44) being less likely to present with obesity. The opposite is true for almost all the older age class categories. Interestingly, after the age of 75, obesity is less likely to be experienced, possibly due to increasing rates of premature mortality [11,40,41]. Being female reduces the probability of the outcome. The civil status also affects the probability of being obese, with unmarried individuals showing a lower likelihood compared to married people, and widowers showing a higher probability. As predicted by the statistical analysis, people living in the south of Italy show a higher probability of being obese. Looking at the lifestyle factors, it is unlikely that current smokers and people practicing sport three days a week are obese.

This picture provides more insights into the characteristics predicting the condition of obesity. Being poor and lower educated, living in the south of Italy, having chronic diseases, and having an age between 55 and 70 years are all characteristics increasing the probability of the outcome. This information may help policymakers in tailoring appropriate measures to reduce the risks of a rise in this condition among the Italian population.

### 3.2. Results from the PSM

The results from the PSM are expressed in terms of the average difference between the group of obese and non-obese individuals, namely the ATT. Table 3 reports the ATT for each model specification, respectively investigating healthcare use, quality of life and the presence of comorbidities. In Section 4, we discuss the results for each category of outcome.

### 3.3. Assessing the Matching Quality

The quality of the PSM was assessed through the balancing t-test; after the matching procedure, we carefully checked the results from the t-test and verified that no statistical difference was left on any covariate between the two groups (results of the t-test available from the authors). For simplicity, we display the main results of the balancing t-test for just one outcome variable, namely, the probability of *experiencing problems in climbing stairs*; the results are almost the same across the three domains. Table 4 shows a significant reduction in the mean and median bias and a zero value for the pseudoR^2^ after matching, providing evidence of the quality of the matching between the treated individuals and controls. The *p* values of the chi square test before and after matching show that a perfect balance is reached after matching and this evidence is corroborated by the Rubin’s R and B values after matching [42]. A further check on the matching quality was provided by the visual inspection of the matching graphs. For each of the outcome variables, the distribution confirms that there is an overlap in the range of propensity scores across the treated and untreated groups.

## 4. Discussion

The present section is dedicated to discussing the results reported in Table 3. We analyze the impact of obesity on the main categories of outcome, specifically healthcare use, quality of life and comorbidities. The strengths and limitations of the research are debated in the last paragraph.

### 4.1. Obesity and Healthcare Use

Our interest lies in detecting a possible increase in the use of healthcare due to the condition of obesity, once several confounding factors are controlled for. Table 3 reports the ATT for each of the selected outcomes representative of healthcare use. With the exception of the use of medicines not prescribed by a GP, the results from the PSM reveal a higher probability of healthcare use by obese individuals compared to non-obese individuals. Specifically, being obese increases by 4.1% the probability of acquiring drugs prescribed by a doctor, while reducing by 1.4% the use of non-prescription drugs. This first evidence is corroborated by the parameters on GP access: obese individuals show a 2.7% higher probability of having visited a GP in the last four weeks compared to their statistical twins. The results hold in sign and significance as the number of GP visits increases, showing higher access to the GP by individuals who are obese. An increment in healthcare use is also shown for diagnostic services (+2%), specialist visits in the last year (+2.1%) and, although very low in value, visits to the psychiatric/psychologist.

We demonstrate that the condition of obesity increases healthcare use for almost all the variables selected, confirming the literature findings [17,19]. Higher access corresponds to higher costs for either the Italian NHS or the patient.

### 4.2. Obesity and Quality of Life

As suggested by the literature, obesity highly affects quality of life [7,13]: it impairs functional status and it affects the cardio circular system, provoking, beyond mortality and morbidity consequences, a constant feeling of fatigue, excessive sweating and negative effects on perceived health [6]. With reference to the psychological sphere, a persistent sense of both inadequateness and low self-esteem characterizes individuals with obesity [8,9,43].

The EHIS provides a set of variables addressing respondents’ attitudes in terms of everyday activities; we employed some of them as outcome variables in our PSM specification, with the aim of testing the eventual differences among obese and non-obese individuals in daily actions. The results from Table 3 show the difficulties of obese people in carrying out ordinary daily actions, such as walking 500 m or performing domestic chores. The highest parameter (+5.8%) relates to problems walking up or down 10 steps, followed by problems walking 500 m (+4.1%). Movement difficulties can highly impair the willingness to go out, the enjoyment of quotidian tasks and also the likeliness of performing working activities, as suggested by the extant literature [9]. The parameter related to receiving help with dailies activities, although low in value (+1%), may signal a problem of scarce autonomy experienced by obese individuals in everyday life.

To further inspect respondents’ quality of life, we also employed questions related to concentration/sleep/energy aspects experienced during the two weeks before the interview. Using a shorter lag time reduces the risk of bias in recalling some aspects of daily activities. The results show that obese individuals are more likely to experience fatigue or less energy (+2.1%), and to show a very low self-esteem (+1.3%). These negative feelings not only boost healthcare expenditure but also impact on individual productivity, increasing the indirect costs of obesity. No evidence was found of the presence of sleep or concentration disorders.

### 4.3. Obesity and Comorbidities

Obesity represents a risk factor for the presence of several diseases, such as cardiovascular diseases, type 2 diabetes mellitus, and cancers. We selected a set of diseases and health conditions addressed by the literature as possible comorbidities related to obesity.

The survey presents the following question: “*Over the last 12 months, did you experience one or more of the following diseases or conditions?*” This question has possible answers of yes or not. For each condition, it is possible to create a binary variable assuming a value of one if the response is positive, and zero otherwise.

The results corroborate the literature findings. The ATT values are especially high for diabetes (+8.9%) and hypertension (+14.3%). Diabetes noticeably affects individuals’ quality of life and involves remarkable healthcare costs for the NHS; hypertension represents a risk for health shocks (e.g., heart attack), with detrimental consequences for individuals’ wellbeing. The results corroborate the empirical findings [4,27] on a higher risk of showing comorbidities by obese individuals compared to non-obese individuals, with consequences for health-related costs. Specifically, a greater likelihood of showing chronic back pain (+5.3%), depression (+2.4%), chronic anxiety (+1.4%) and other comorbidities is shown. The probability of experiencing a heart attack is slightly significant and very low in value, while there is evidence (+1.7%) of presenting other chronic heart diseases.

### 4.4. Strengths and Limitations of the Research

The analysis has some limitations related to data availability. First of all, it was not possible to provide a precise estimate of the economic impact related to healthcare access, since the identification of healthcare services is too generic to be converted into monetary values; for example, the results show that obese people consume more pharmaceutical products compared to their non-obese statistical twins, although the name of the drug is not revealed in the database and consequently it is not possible to quantify the pharmaceutical expenditure. Likewise, the data does not allow us to distinguish between the costs sustained by the NHS and those covered by the patients themselves. The Italian NHS provides full coverage for the whole population, but patients can also opt for private access and pay the full price. The data reported in Table 3 shows that obese people consume more specialist and psychiatric visits, as well as more diagnostic care, but information on whether the access is private or covered by the NHS is not available. Disentangling this question would have provided a clearer frame on the financial burden of obesity. However, we clearly show a higher consumption of healthcare services associated with obesity, which corresponds to increasing costs for both the NHS and patients and raises concern about the economic burden of this disease. Among the limits, we could not distinguish the three levels of obesity. This information would have provided more insight into this condition, allowing us to stratify the outcomes according to the category of obesity.

Despite the limitations, our study responds to an important information gap, providing novel evidence on the phenomenon of obesity in Italy and addressing relevant policy implications. Specifically, the analysis carried out has the merit of using microdata representative of the Italian population, which allows us to thoroughly investigate features related to obese individuals in terms of healthcare access, quality of life and the presence of comorbidities. The inspection of the socioeconomic determinants of this condition completes the analysis, raising concern about possible inequalities related to the spread of obesity across the Italian population. The analysis corroborates evidence from the literature and shows an updated frame of obesity in Italy, suggesting the implementation of new measures to prevent and contrast the increasing trend of obesity in Italy.

## 5. Conclusions

Obesity is a multifaceted condition involving significant costs for both patients and society. In Italy, the phenomenon is increasing and includes 11.1% of the population, according to the EHIS 2019 [20].

Although this condition has been accurately analyzed from different perspectives worldwide, in Italy, due to the relatively recent spread of obesity, a broad vision of its distribution across socioeconomic classes and of its effects on population health and wellbeing is missing. We found evidence of socioeconomic determinants in the distribution of obesity, with the richest and more educated individuals being less likely to present with this condition. Other risk factors are age, gender and macro-region of residence, with the population from southern regions more exposed to this condition.

The use of PSM allows deeper insight into the impact of obesity on selected outcomes, namely healthcare use, quality of life and the presence of other comorbidities. The results show that obese individuals consume more healthcare services compared to individuals with the same observable characteristics but for obesity. For example, obese individuals present a 3% higher probability of having visited a GP in the last four weeks, a 2% higher probability of access to diagnostic services, and a 4% higher probability of having consumed drugs prescribed by a doctor. This different consumption pattern provides insightful information in terms of the healthcare costs attributable to the obese population in Italy. Likewise, this condition implies a higher probability of experiencing problems in daily activities, such as walking for 500 m (+6%) or climbing stairs (+4%). Other physical and psychological aspects are detected, such as less energy or low self-esteem, which highly impair the quality of life of obese individuals, with consequences for both the direct and indirect costs. Finally, being obese represents a risk factor for the presence of comorbidities, especially diabetes (+9%) or other chronic conditions (hypertension and/or back pain), enlarging the healthcare costs attributed to this disease and raising concern about the healthcare expenditure in the years to come.

The increasing trend of obesity across Italian population calls for prevention measures focused on the importance of appropriate lifestyles, such as eating, consumption habits and physical activity. We found evidence of higher access to the GP by the obese population and we recognize the central role of this actor in monitoring and informing patients, especially those in low socioeconomic conditions. To avoid an excessive workload for GPs, an appropriate clinical pathway should be implemented across the Italian regions, involving different levels of care: GPs should play the role of case manager, community nurses could support them by both monitoring patients’ lifestyles and controlling for the eventual presence of comorbidities, while specialists should be involved in the most severe case. A global design, encompassing stakeholders within shared healthcare tracks, could help in reducing this increasing phenomenon.

Any other governmental action preventing obesity or warning about its consequences (e.g., information campaign on lifestyle) should be prioritized. Diverse channels should be employed to inform the population, especially individuals in low socioeconomic classes, of the possible risks associated to obesity. Educational campaigns can be implemented at schools, at working places, or through the mass media and social networks. At the regional or community level, some possible measures are represented by subsidies for physical activity programs, promotion of wellness programs for selected categories (e.g., pensioners), and discounts on selected food (e.g., fruit and vegetable once a week). All these measures would help in reducing the economic burden of obesity and decreasing the inequalities caused by socioeconomic factors.

## Figures and Tables

**Table 1:** Outcome variables—descriptive statistics and variable definitions—standard deviation in parenthesis.

Variable Name	Variable Definition	Full Sample *n* = 44,902	Obese Individuals (Treated) *n* = 4951	Non-Obese Individuals (Untreated) *n* = 39,951
		Mean	Mean	Mean
Healthcare consumption				
Drugs prescribed by a doctor	Use of drugs prescribed by a doctor in the last two weeks	0.396 (0.489)	0.538 (0.499)	0.379 (0.485)
Drugs not prescribed by a doctor	Use of drugs not prescribed by a doctor in the last two weeks	0.599 (0.490)	0.459 (0.498)	0.616 (0.486)
GP 1 visit *	One visit to the GP in the last 4 weeks	0.207 (0.405)	0.246 (0.431)	0.202 (0.401)
GP 2 or more visits	Two or more visits to the GP in the last 4 weeks	0.106 (0.308)	0.174 (0.379)	0.098 (0.297)
GP 3 or more visits	Three or more visits to the GP in the last 4 weeks	0.039 (0.193)	0.071 (0.257	0.035 (0.184)
Specialist visit	At least 1 access to a specialist in the last four weeks	0.182 (0.386)	0.230 (0.421)	0.176 (0.381)
Specialist in the last year	At least 1 access to a specialist in the last 12 months	0.552 (0.497)	0.623 (0.485)	0.544 (0.498)
Psychiatric psychologist	At least 1 access to a psychiatrist/psychologist in the last year	0.037 (0.188)	0.048 (0.214)	0.035 (0.184)
Diagnostic services	Performing at least one diagnostic test in the last 12 months	0.415 (0.493)	0.486 (0.500)	0.406 (0.491)
Comorbidities				
Diabetes	Presence of diabetes in the last twelve months	0.071 (0.256)	0.179 (0.383)	0.057 (0.232)
Hypertension	Presence of hypertension in the last twelve months	0.220 (0.414)	0.422 (0.494)	0.195 (0.396)
Chronic back pain	Presence of chronic back pain in the last twelve months	0.193 (0.394)	0.301 (0.459)	0.179 (0.384)
Depression	Presence of depression in the last twelve months	0.059 (0.236)	0.105 (0.307)	0.053 (0.225)
Chronic anxiety	Presence of chronic anxiety in the last twelve months	0.041 (0.199)	0.070 (0.255)	0.038 (0.191)
Heart attack	Experiencing a heart attack in the last twelve months	0.019 (0.137)	0.035 (0.183)	0.017 (0.131)
Chronic heart disease	Presence of chronic heart diseases in the last twelve months	0.053 (0.225)	0.092 (0.289)	0.049 (0.215)
Chronic bronchitis	Presence of bronchitis in the last twelve months	0.048 (0.214)	0.092 (0.289)	0.043 (0.202)
Quality of life				
Domestic chores difficulties	Some or high difficulties in doing domestic chores	0.094 (0.291)	0.147 (0.354)	0.087 (0.282)
Stair difficulties	Difficulties in walking up or down 10 steps—some to high problems	0.122 (0.327)	0.233 (0.423)	0.108 (0.310)
Walking difficulties	Walking 500 m—some to high problems	0.106 (0.307)	0.197 (0.398)	0.094 (0.292)
Receiving help	Receiving help from other people in dailies activities/using devices (sticks, wheelchair, etc.)	0.054 (0.227)	0.095 (0.293)	0.049 (0.216)
Concentration difficulties	Difficulty in concentration during the last two weeks—some to very high problems	0.176 (0.381)	0.236 (0.425)	0.168 (0.374)
Sleep disorders	Experiencing sleep disorders in the last two weeks	0.283 (0.450)	0.348 (0.476)	0.275 (0.447)
Less energy	Experiencing fatigue or less energy during the last two weeks—half times and ever	0.449 (0.497)	0.531 (0.499)	0.439 (0.496)
Low self esteem	Having a low opinion of herself. feeling like a failure—sometimes to very often	0.108 (0.311)	0.145 (0.352)	0.104 (0.305)

* GP: general practitioner. Source: Our elaboration based on the EHIS, 2019 [20].

**Table 2:** Control variables—descriptive statistics and variable definitions—standard deviation in parenthesis.

Variable Name	Variable Definition	Full Sample *n* = 44,902	Obese Individuals (Treated) *n* = 4951	Non-Obese Individuals (Untreated) *n* = 39,951
		Mean	Mean	Mean
Obesity condition	Being obese	0.110 (0.313)	1.000 (0.000)	0.000 (0.000)
Income quintiles	Income quintiles from poorest to richest	3.080 (1.403)	2.970 (1.393)	3.09 (1.404)
Limitation high	Presence of severe limitations	0.078 (0.268)	0.140 (0.347)	0.070 (0.256)
Chronic disease	Presence of 1 or more chronic diseases	0.325 (0.468)	0.500 (0.500)	0.303 (0.459)
Bad health	Declaring bad and very bad health	0.084 (0.278)	0.153 (0.360)	0.076 (0.265)
Fine health	Declaring not bad not good health	0.227 (0.419)	0.328 (0.470)	0.214 (0.410)
Good health (omitted)	Declaring good and very good health	0.688 (0.463)	0.519 (0.500)	0.709 (0.454)
GP visit	At least one visit to the general practitioner in the last 4 weeks	0.313 (0.464)	0.420 (0.494)	0.300 (0.458)
SPC visit	At least one visit to a specialist in the last four weeks	0.182 (0.386)	0.230 (0.421)	0.176 (0.381)
Long waiting times	Experiencing long waiting time for healthcare access	0.140 (0.347)	0.184 (0.387)	0.135 (0.342)
Work unemployed	Being unemployed	0.081 (0.273)	0.066 (0.248)	0.083 (0.276)
Work employed (omitted)	Being employed	0.428 (0.495)	0.378 (0.485)	0.434 (0.496)
Work retired	Being retired	0.245 (0.430)	0.333 (0.471)	0.234 (0.424)
Work other	Other working activities, e.g., student, homemaker	0.246 (0.431)	0.223 (0.416)	0.249 (0.432)
School low	Finishing lower secondary school	0.477 (0.499)	0.605 (0.489)	0.461 (0.498)
School high (omitted)	Finishing high school	0.368 (0.482)	0.3 (0.458)	0.376 (0.484)
School un	Holding university or post-university degree	0.155 (0.362)	0.096 (0.294)	0.163 (0.369)
Age15_17	Age class 15 to 17	0.031 (0.172)	0.003 (0.051)	0.034 (0.181)
Age18_24	Age class 18 to 24	0.074 (0.262)	0.018 (0.131)	0.081 (0.273)
Age25_34	Age class 25 to 34	0.101 (0.301)	0.052 (0.221)	0.107 (0.309)
Age35_44 (omitted)	Age class 35 to 44,	0.137 (0.343)	0.109 (0.312)	0.14 (0.347)
Age45_49	Age class 45 to 49	0.090 (0.286)	0.095 (0.294)	0.089 (0.285)
Age50_54	Age class 50 to 54	0.096 (0.295)	0.102 (0.302)	0.096 (0.294)
Age55_59	Age class 55 to 59	0.089 (0.285)	0.113 (0.316)	0.086 (0.281)
Age60_64	Age class 60 to 64	0.081 (0.274)	0.104 (0.305)	0.079 (0.269)
Age65_69	Age class 65 to 69	0.077 (0.266)	0.116 (0.320)	0.072 (0.259)
Age70_74	Age class 70 to 74	0.072 (0.258)	0.110 (0.313)	0.067 (0.25)
Age75_Over	Age class 75 or more	0.152 (0.359)	0.180 (0.384)	0.149 (0.356)
Female	Being female	0.526 (0.499)	0.493 (0.500)	0.530 (0.499)
Single	Being unmarried	0.304 (0.460)	0.172 (0.378)	0.320 (0.466)
Married (omitted)	Being married	0.547 (0.498)	0.634 (0.482)	0.536 (0.499)
Widower	Being widower	0.098 (0.297)	0.137 (0.344)	0.093 (0.290)
Divorced	Being divorced	0.052 (0.222)	0.056 (0.230)	0.051 (0.221)
Geo south islands	Living in southern regions and islands	0.372 (0.483)	0.408 (0.491)	0.368 (0.482)
Geo center	Living in central regions	0.191 (0.393)	0.173 (0.379)	0.193 (0.394)
Geo north	Living in northern regions	0.437 (0.496)	0.419 (0.493)	0.439 (0.496)
Health ins yes	Having an integrative insurance	0.151 (0.358)	0.125 (0.331)	0.154 (0.361)
Smoke curr	Being a current smoker	0.163 (0.370)	0.146 (0.353)	0.165 (0.372)
Sport 3 days	Practicing sport three days a week	0.195 (0.396)	0.118 (0.323)	0.205 (0.404)

Source: Our elaboration based on the EHIS, 2019 [20].

**Table 3:** ATT between obese and non-obese individuals on healthcare use, quality of life and presence of comorbidities—standard errors in parenthesis.

Healthcare Use		Quality of Life		Presence of Comorbidities	
Variable of Outcome	ATT Radius (cal. 0.01)	Variable of Outcome	ATT Radius (cal. 0.01)	Variable of Outcome	ATT Radius (cal. 0.01)
Drugs prescribed by a doctor	0.041 *** (0.008)	Domestic chores	0.017 *** (0.005)	Diabetes	0.089 *** (0.006)
Drugs not prescribed by a doctor	−0.014 *** (0.006)	Stair difficulties	0.058 *** (0.006)	Hypertension	0.143 *** (0.007)
GP yes	0.027 *** (0.007)	Walking difficulties	0.041 *** (0.006)	Chronic back pain	0.053 *** (0.007)
GP access two or more	0.021 *** (0.006)	Receiving help	0.01 *** (0.004)	Depression	0.024 *** (0.005)
GP access three or more	0.012 *** (0.004)	Concentration difficulty ^1^	−0.005 (0.006)	Chronic anxiety	0.014 *** (0.004)
Specialist visit	0.001 * (0.006)	Sleep disorders ^1^	0.01 (0.007)	Heart attack	0.004 * (0.003)
Specialists in the last year	0.021 *** (0.007)	Less energy ^1^	0.021 *** (0.008)	Chronic heart diseases	0.017 *** (0.004)
Psychiatric psychologist	0.005 ** (0.003)	Low self-esteem ^1^	0.013 *** (0.005)	Chronic bronchitis	0.024 *** (0.004)
Diagnostic services	0.02 *** (0.008)				

ATT: Average treatment effect on the treated. ^1^ Time lag: two weeks before the questionnaire *** *p* < 0.01; ** *p* < 0.05; * *p* < 0.1. Source: Our elaboration based on the EHIS, 2019 [20].

**Table 4:** Balance test results of the PSM—outcome variable: problems in climbing stairs.

Sample	Pseudo R^2^	*p* > chi^2^	Mean Bias	Median Bias	B	R
Unmatched	0.062	0.000	15.7	13.7	68.8	0.60
Matched	0.000	1.000	0.3	0.3	2.8	1.05

Source: Our elaboration based on the EHIS, 2019 [20].

## Data Availability

EHIS (European Health Interview Survey) 2019, Italy [20]. Available online: https://www.istat.it/en/non-categorizzato/european-health-interview-survey-ehis/ (accessed on 27 April 2025).
